# Photocatalytic Degradation of Organic Dyes by Magnetite Nanoparticles Prepared by Co-Precipitation

**DOI:** 10.3390/ijms25147876

**Published:** 2024-07-18

**Authors:** Thandi B. Mbuyazi, Peter A. Ajibade

**Affiliations:** School of Chemistry and Physics, University of KwaZulu-Natal, Private Bag X01, Scottsville, Pietermaritzburg 3209, South Africa

**Keywords:** iron oxide, nanoparticles, photocatalytic degradation, organic dyes, scavenger activity

## Abstract

Iron oxide nanoparticles were synthesized by co-precipitation using three different iron salt stoichiometric mole ratios. Powder X-ray diffraction patterns revealed the inverse cubic spinel structure of magnetite iron oxide. Transmission electron microscopic images showed Fe_3_O_4_ nanoparticles with different shapes and average particle sizes of 5.48 nm for Fe_3_O_4_-**1:2**, 6.02 nm for Fe_3_O_4_-**1.5:2**, and 6.98 nm for Fe_3_O_4_-**2:3** with an energy bandgap of 3.27 to 3.53 eV. The as-prepared Fe_3_O_4_ nanoparticles were used as photocatalysts to degrade brilliant green (BG), rhodamine B (RhB), indigo carmine (IC), and methyl red (MR) under visible light irradiation. The photocatalytic degradation efficiency of 80.4% was obtained from Fe_3_O_4_-**1:2** for brilliant green, 61.5% from Fe_3_O_4_-**1.5:2** for rhodamine B, and 77.9% and 73.9% from Fe_3_O_4_-**2:3** for both indigo carmine and methyl red. This indicates that Fe_3_O_4_-**2:3** is more efficient in the degradation of more than one dye. This study shows that brilliant green degrades most effectively at pH 9, rhodamine B degrades best at pH 6.5, and indigo carmine and methyl red degrade most efficiently at pH 3. Recyclability experiments showed that the Fe_3_O_4_ photocatalysts can be recycled four times and are photostable.

## 1. Introduction

Azo dyes are the most prevalent and biggest group of artificial dyes that are extensively used in the textiles, beauty products, pharmaceutical, and food industries [1,2]. The use of azo dyes has seen a dramatic increase in the textile industry over the past decades for coloring cotton and wool because of their vibrant colors, affordability, and excellent durability upon exposure to light and washing [3]. Most of these dyes have mutagenic, cancer-causing, genotoxic, and teratogenic properties [4]. Furthermore, their presence in water bodies inhibits solar radiation from penetrating, lowering oxygen quantity, interfering with photosynthesis, and damaging terraqueous ecosystems [5,6]. As a result, researchers have long been involved in developing an efficient, cost-effective, and dependable method for eliminating dyes from wastewater [7,8].

Photodegradation is commonly used to remove contaminants from the environment [9]. It involves subjecting pollutants to light, which causes their photochemical breakdown into simpler, non-toxic compounds. This method is frequently preferred due to its economic viability, sustainability, absence of secondary waste, and broad-spectrum degradation of different organic dyes into CO_2_ and H_2_O [10,11]. The practicality of the procedure can be improved by using a photocatalyst, which is a semiconducting material that is excited by absorbing light photons. Iron oxide is one of the materials that show potential for photocatalytic applications because of its cost-effective synthesis, magnetic properties, and n-type semiconducting effects [12].

Previous studies have shown the properties of superparamagnetic iron oxide nanoparticles synthesized by co-precipitation could be influenced by variations of the preparative parameters [3,13,14]. Alibeigi and Vaezi investigated the effect of Fe^2+^/Fe^3+^ molar ratios and observed that increasing the iron salt ratio increases crystallinity and specific saturation magnetization [15]. A study by Dehghanpour also demonstrated that adjusting the mole ratio of Fe^2+^ to Fe^3+^ ions, pH, and capping agents may influence saturation magnetization and the size of the nanoparticles [16]. Another study reported that changing the stoichiometric ratio of 1:2 for Fe^2+^ and Fe^3+^ salts decreased electrical conductivity and led to the phase change of the nanoparticles from Fe_3_O_4_ to γ-Fe_2_O_3_ [17]. Iida et al. [18] synthesized Fe_3_O_4_ nanoparticles by hydrolysis in an aqueous solution comprising ferrous and ferric salts in varying ratios, with 1,6-hexane-diamine as the base. An increase in the ratio of Fe^2+^ to Fe^3+^ ions resulted in an increase in the size of Fe_3_O_4_ nanoparticles from 9 to approximately 37 nm. Lee et al. [19] studied the antioxidant properties and physiological effects of maghemite nanoparticles (γ-Fe_2_O_3_) prepared by varying Fe^2+^/Fe^3+^ molar ratios using ferrous/ferric salt co-precipitation. Thus, studies have shown that the physical and chemical changes that occur in iron oxide nanoparticles when the Fe^2+^/Fe^3+^ molar ratio varies throughout the co-precipitation process. However, no work has studied the photocatalytic potential of Fe_3_O_4_ with respect to different Fe^2+^ to Fe^3+^ molar ratios.

The surface properties, crystalline structure, and morphology of the nanoparticles, which are all controlled by the synthetic technique, could influence the photocatalytic degradation efficiency of magnetite nanoparticles [20]. Studies have shown that a change in dye pH, light source, and surface charge influences the photocatalytic activity of Fe_3_O_4_ nanoparticles against organic dyes such as rhodamine B [21], methyl red [22], brilliant green [23], and indigo carmine [24]. In the current study, we evaluate the influence of the Fe^2+^/Fe^3+^ salt ratios on the structural and morphological optical and photocatalytic properties of Fe_3_O_4_ nanoparticles synthesized via the co-precipitation method. The potential of the as-synthesized iron oxide nanoparticles as photocatalysts for the photodegradation of brilliant green, rhodamine B, indigo carmine, and methyl red under visible light was studied. Additionally, the photostability of the nanoparticles, the effect of pH, and scavengers on the photodegradation of the organic dyes by the iron oxide nanoparticles were evaluated.

## 2. Results

### 2.1. Morphological Studies of the Iron Oxide Nanoparticles

**Figure 1:**
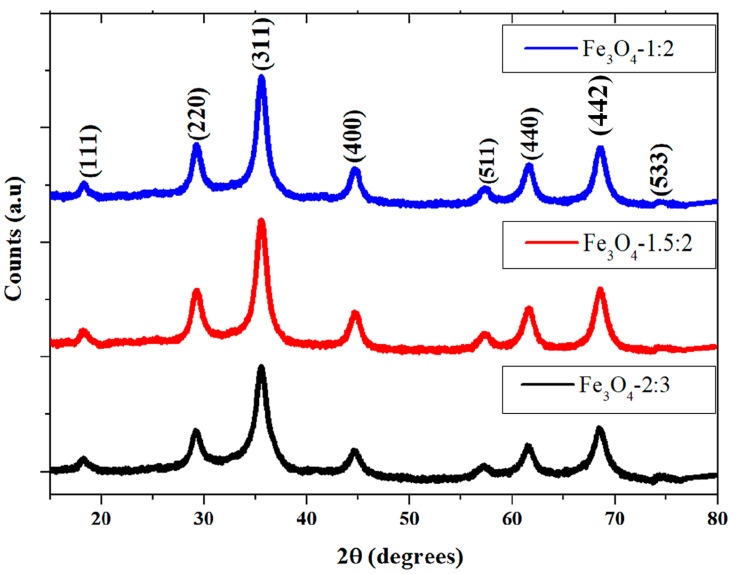
XRD patterns of Fe_3_O_4_ nanoparticles were prepared using different iron salt stochiometric ratios.

Figure 1 displays the diffractograms of the iron oxide nanoparticles prepared using different iron salt ratios. The 2θ peaks observed at 18.23°, 29.31°, 35.73°, 44.66°, 57.27°, 61.69°, 68.65, and 74.38° correlate to (111), (220), (311), (400), (511), (440), (442), and (533) reflections of inverse cubic spinel structure of magnetite (JCPDS no: 19-0629) [25,26]. The crystal growth direction inclination is toward the (311) plane. The diffraction patterns suggest that the iron salt ratios used have no apparent impact on the crystalline phases of the as-synthesized iron oxide nanoparticles. 

The average crystallite sizes of the magnetite nanoparticles were calculated using the Debye–Scherrer equation [27]:

(1) D=Kλ/βcosθ

where *D*, *K*, *λ*, *β*, and *θ* stand for crystallite diameter (nm), Scherrer constant, X-ray wavelength, full width at half-maximum (FWHM), and Bragg angle, respectively. The FWHM and *θ* were calculated by fitting all diffraction peaks to a Gaussian function. The calculated crystallite sizes were 4.45 nm, 5.89 nm, and 6.50 nm for Fe_3_O_4_-**1:2**, Fe_3_O_4_-**1.5:2**, and Fe_3_O_4_-**2:3**, respectively. The lattice parameter is another significant piece of information that may be retrieved from XRD patterns. The lattice parameter of magnetite nanoparticles was derived using Bragg’s rule and the following equation [28].

(2) $$a=d_{hkl}\sqrt{h^{2}+k^{2}+l^{2}}$$

where *a*, *d_hkl_*, and *hkl* are the lattice constant, inter-planer distance, and Miller indices, respectively. Magnetite (JCPDS 19-0629) has a standard lattice constant of 8.3960 Å. The calculated lattice constants of the as-prepared magnetite nanoparticles are 8.3686 Å, 8.3579 Å, and 8.3574 Å for Fe_3_O_4_-**1:2**, Fe_3_O_4_-**1.5:2**, and Fe_3_O_4_-**2:3**, respectively. The results show that the calculated lattice constants of the as-prepared magnetite nanoparticles slightly vary from one another, and they are less than that of standard magnetite. This discrepancy could be attributed to the effect of various crystal size variations on the crystal lattice [29].

**Figure 2:**
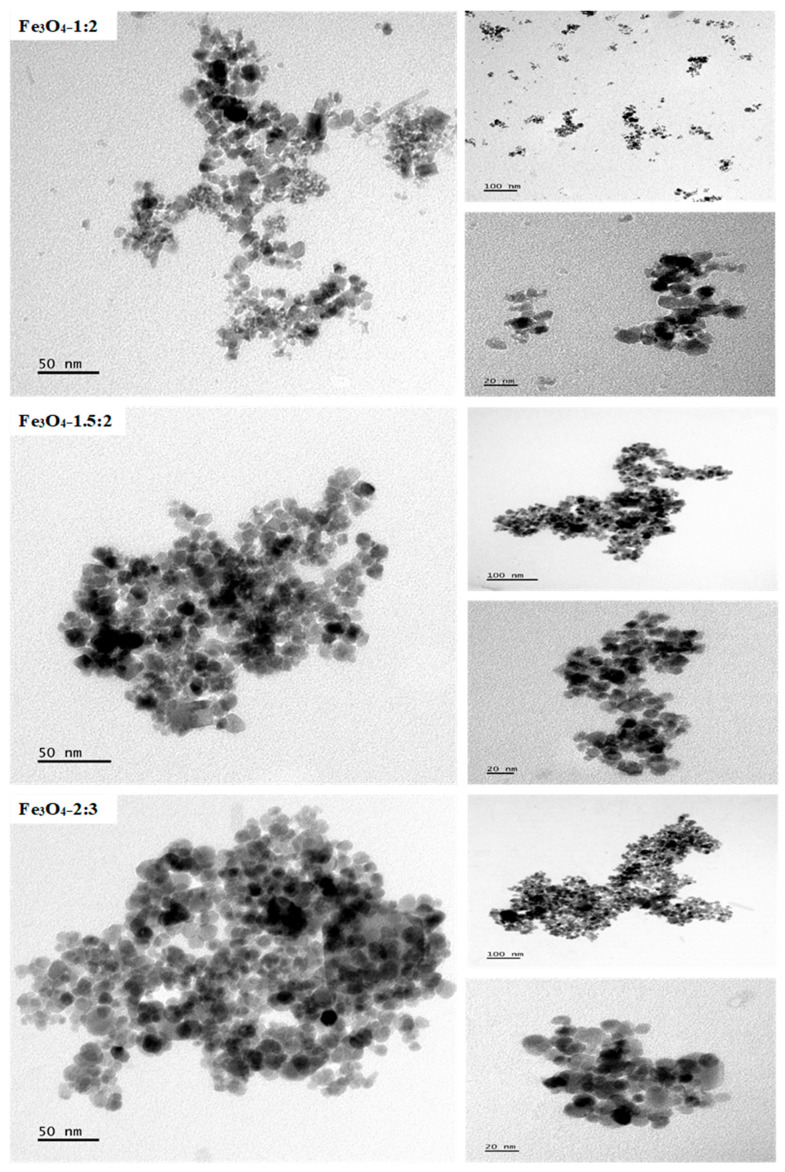
TEM images of Fe_3_O_4_-**1:2**, Fe_3_O_4_-**1.5:2**, and Fe_3_O_4_-**2:3** nanoparticles at different magnifications.

TEM images in Figure 2 revealed that Fe_3_O_4_-**1:2** nanoparticles are a combination of square-like, oval, rod, and rectangular shapes with a mean size of 5.48 nm, while Fe_3_O_4_-**1.5:2** nanoparticles are agglomerated square-like, rectangular, and oval-shaped with a mean size of 6.02 nm. Fe_3_O_4_-**2:3** nanoparticles are also agglomerated and quasi-spherical, with an average size of 6.98 nm. There is no drastic change in average particle size with an increase in the stoichiometric ratios of the iron salts, as reported in other studies [16,18]. However, there is apparent agglomeration for Fe_3_O_4_-**1.5:2** and Fe_3_O_4_-**2:3** nanoparticles and larger particle size distributions compared to Fe_3_O_4_-**1:2** (Figure S1).

FTIR was used to study the interactions between octylamine and the as-synthesized Fe_3_O_4_ nanoparticles (Figure S2). The weak ν(N–H)_asy_ and ν(N–H)_sym_ vibrations at 3376 and 3291 cm^−1^ are assigned to the amine group of the capping agent [30]. The C–H characteristic peaks of the octylamine alkyl chain are at 2919 and 2851 cm^−1^ [31]. The C–N bending vibrations appear at 1460 cm^−1^. The capping agent vibrational bands observed also appear in the spectra of the iron oxide nanoparticles but with low intensity, which suggests the presence of an octylamine capping agent on the surface of iron oxide nanoparticles. However, the N–H bands were not observed in the Fe_3_O_4_ nanoparticles spectra, signifying that the capping of the nanoparticles took place through the -NH_2_ group of octylamine [32]. Furthermore, the Fe–O peak at 550 cm^−1^ confirms the synthesis of iron oxide nanoparticles [33].

### 2.2. Optical Studies of the Magnetite Nanoparticles

**Figure 3:**
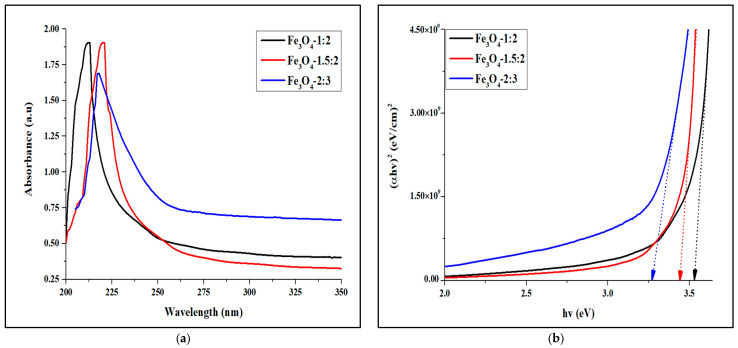
(**a**) Fe_3_O_4_ nanoparticles absorption spectra and (**b**) Tauc plots.

Figure 3a displays the UV-Vis spectra of the as-prepared iron oxide nanoparticles, which exhibit sharp absorption peaks at 212.5 nm, 220.4, and 217.6 nm for Fe_3_O_4_-**1:2**, Fe_3_O_4_-**1.5:2**, and Fe_3_O_4_-**2:3**, respectively. To further investigate the optical properties of the magnetite nanoparticles, the Tauc plot was used to calculate the energy gap [34]. The estimated energy gaps were 3.53 eV for Fe_3_O_4_-**1:2**, 3.44 eV for Fe_3_O_4_-**1.5:2**, and 3.27 eV for Fe_3_O_4_-**2:3** (Figure 3b). The bandgap is observed to decrease with an increase in iron salt ratios, with Fe_3_O_4_-**2:3** nanoparticles having a larger average particle size and the smallest bandgap. The energy gap of the as-synthesized magnetite nanoparticles is greater than that of bulk magnetite (0.20 eV) due to the quantum confinement effect [35]. The optical bandgap of Fe_3_O_4_ nanoparticles has been reported to range from 3.23 to 3.9 eV [36,37]. Moreover, Shamaila et al. synthesized Fe_3_O_4_ nanoparticles with an energy bandgap of 2.5 eV and titanium-coated Fe_3_O_4_ nanoparticles with a bandgap of 3.22 eV [38]. The bare Fe_3_O_4_ demonstrated poor photocatalytic activity, whereas the titanium-coated Fe_3_O_4_ exhibited improved performance under visible light. The optical bandgaps of the magnetite nanoparticles in this study are comparable to previously reported bandgaps [36,37,38], and the nanoparticles have the potential to be used as photocatalysts for dye degradation under visible light.

### 2.3. Surface Properties

**Figure 4:**
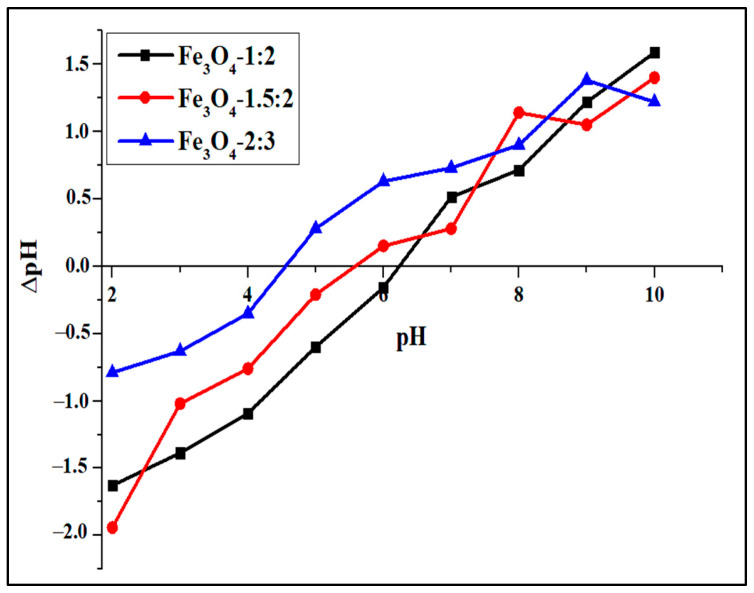
pHpzc of magnetite nanoparticles.

The point of zero charge (PZC) of the magnetite nanoparticles was determined, as shown in Figure 4. The pH_pzc_ values of Fe_3_O_4_-**1:2**, Fe_3_O_4_-**1.5:2**, and Fe_3_O_4_-**2:3** were found to be 6.22, 5.56, and 4.55, respectively. The pH_pzc_ values obtained in this study are comparable to some pH_pzc_ values for magnetite found in the literature [39,40]. At the PZC, the catalyst surface consists of an equal proportion of positive and negative functional groups, yielding a zero net charge. The surface accumulates a negative charge above the PZC and a positive charge below the PZC. This is due to the presence of FeOH groups on the surface of magnetite iron oxide nanoparticles in aqueous solution [41]. These hydroxyl groups can protonate or deprotonate to produce FeOH_2_^+^ or FeO^−^, which changes in response to pH values below or above the pH_PZC_.

## 3. Adsorption Studies of Magnetite Nanoparticles

**Figure 5:**
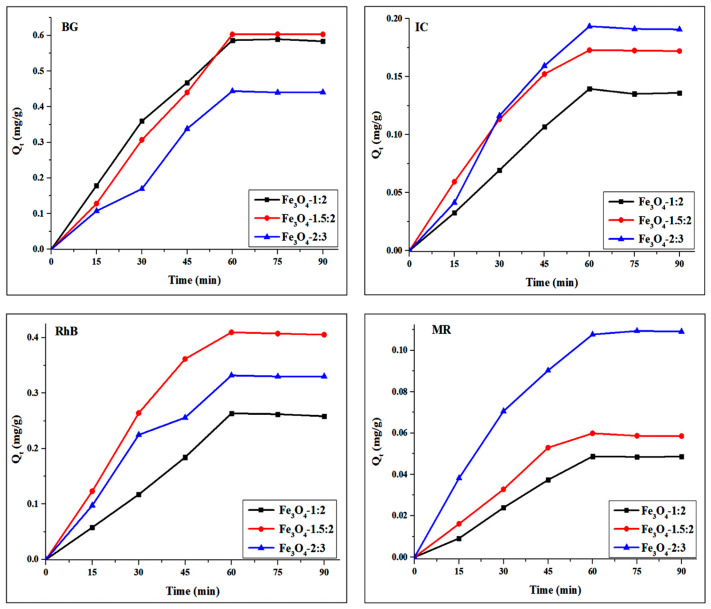
Adsorption capacity of Fe_3_O_4_ nanoparticles.

The adsorption capacity of Fe_3_O_4_ nanoparticles over BG, RhB, IC, and MR dyes was evaluated. The impact of contact time on dye adsorption on Fe_3_O_4_ nanoparticles was investigated to determine the equilibrium interval (Figure 5). Significant adsorption efficiency was noted during the first 45 min, indicating the availability of accessible sites [42,43]. Maximum adsorption capacity was attained at 60 min; beyond this time, adsorption remained constant. Therefore, a 60 min equilibration time was decided upon for the adsorption period prior to visible light exposure for the photocatalytic studies.

## 4. Photocatalytic Studies of Magnetite Nanoparticles

**Figure 6:**
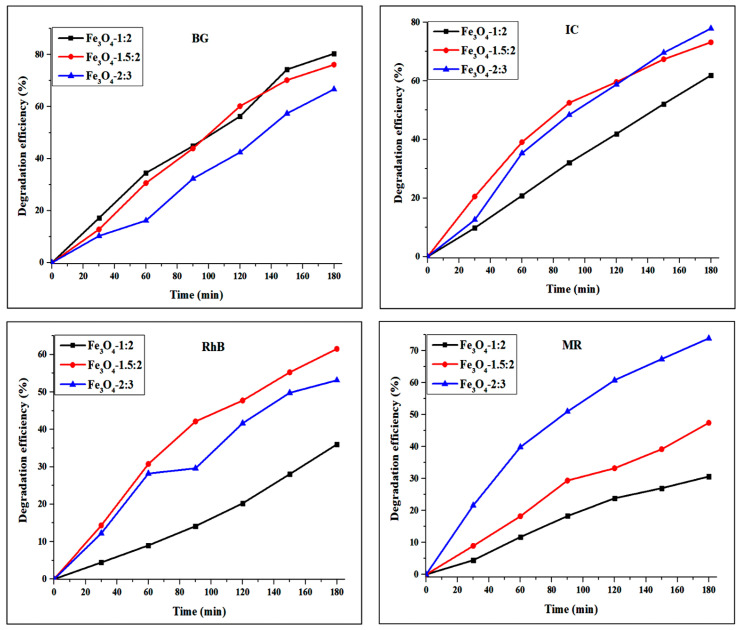
Degradation efficiency plots of BG, RhB, IC, and MR by Fe_3_O_4_ nanoparticles.

The photocatalytic degradation efficiencies of the Fe_3_O_4_ nanoparticles were evaluated using BG, RhB, IC, and MR under visible light (Figure 6). The percentage degradation of BG after 180 min irradiation is 80.4%, 76.1%, and 66.7% by Fe_3_O_4_-**1:2**, Fe_3_O_4_-**1.5:2**, and Fe_3_O_4_-**2:3**, respectively. The degradation efficiency of 36.0%, 61.5%, and 53.1% was obtained for Fe_3_O_4_-**1:2**, Fe_3_O_4_-**1.5:2**, and Fe_3_O_4_-**2:3**, respectively, against RhB dye. Different degradation efficiencies may be attributed to the morphologies of the iron oxide nanoparticles. The low percentage degradation of RhB by the as-prepared iron oxide nanoparticles could be ascribed to the dye’s intricate structure [44]. The photocatalytic degradation efficiencies of IC by Fe_3_O_4_-**1:2**, Fe_3_O_4_-**1.5:2**, and Fe_3_O_4_-**2:3** was 61.9%, 73.2%, and 77.9%. Meanwhile, the efficiencies of 30.6%, 47.4%, and 73.9% were obtained for Fe_3_O_4_-**1:2**, Fe_3_O_4_-**1.5:2**, and Fe_3_O_4_-**2:3** against MR dye, respectively. The results show that Fe_3_O_4_-**2:3** exhibited high degradation efficiency for both IC and MR. The enhanced efficiency of Fe_3_O_4_-**2:3** nanoparticles can be ascribed to their small particle size, leading to a greater surface area that facilitates more interactions with dye molecules. It has been demonstrated that size is a critical factor influencing the activity of photocatalysts. The catalyst’s size and shape affect its surface structure, which subsequently influences its interaction with dye molecules [45,46].

The comparisons between the current work and previous studies on the degradation of BG, IC, MR, and RhB dyes are presented in Table 1. In most studies, doped or supported nanoparticles are used under UV light or high-wattage visible light. However, in this study, high degradation efficiency was achieved using a light source with lower wattage and unsupported magnetite nanoparticles. The high degradation efficiency of the magnetite nanoparticles may be attributed to the presence of both Fe^2+^ and Fe^3+^ ions that can facilitate the generation of ·OH radicals, improving the photocatalytic reaction [47].

The photodegradation rate constants of organic dyes by the as-prepared Fe_3_O_4_ nanoparticles were further studied using the Langmuir–Hinshelwood pseudo-first-order kinetic model [59,60]. The reaction rate constants were calculated from the gradient of the ln(C_t_/C_0_) versus time plot (Figure S3) and presented in Table 2. The photodegradation rate constants were calculated to be in the range of 0.0061–0.0091 min^−1^ for BG, 0.0024–0.0053 min^−1^ for RhB, 0.0053–0.0084 min^−1^ for IC, and 0.0021–0.0074 min^−1^ for MR. In accordance with the degradation efficiency curves, the Fe_3_O_4_-**2:3** degradation rate constant was higher for anionic dyes but lower for cationic dyes. The correlation coefficient (R^2^) values of <0.97 indicate that the photocatalytic decomposition of all dyes fitted the pseudo-first-order model [61].

### 4.1. Effect of Scavengers on the Degradation of Organic Dyes by Fe_3_O_4_ Nanoparticles

**Figure 7:**
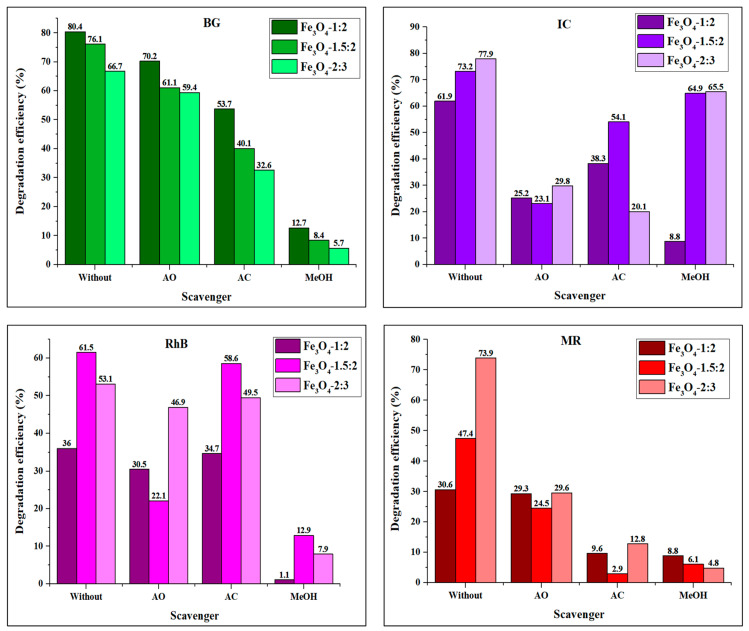
Effect of scavengers on the photocatalytic degradation of brilliant green (BG), indigo carmine (IC), rhodamine B (RhB), and methyl red (MR) by Fe_3_O_4_ nanoparticles.

The photocatalytic degradation reaction is regulated by a movement of electron–hole pairs photogenerated on the surface of the catalyst. Therefore, it is crucial to establish the effect of the reactive species in the photodegradation reaction [62,63]. Hence, methanol alcohol, acrylamide, and ammonium oxalate were introduced as scavengers to quench ·OH, ·O_2_^−^, and h^+^ in the photocatalytic reaction, respectively. Figure 7 illustrates the percentage degradation of the organic dyes when different scavengers are added to the reaction system. The bar graphs show that the photodegradation efficiency of Fe_3_O_4_ nanoparticles was significantly reduced upon the introduction of methanol (MeOH), which suggests that ·OH plays a major part in the degradation of BG, RhB, and MR. The photodegradation efficiency of BG decreased to 32.6–53.7% and 59.4–70.2% after adding AC and AO, respectively. This indicates that ·OH and ·O_2_^−^ are the major active species in the degradation of BG, with h^+^ serving as a secondary species. This trend is consistent with the literature [64,65]. Adding MeOH and AO inhibited the photodegradation of rhodamine B drastically, indicating that h^+^ and ·OH are the major active species, while ·O_2_^−^ plays a limited part in the photodegradation process [66,67]. For indigo carmine, the order of reactive oxidative species responsible for photocatalytic degradation by the Fe_3_O_4_ was different for all nanoparticles. The observed trend for Fe_3_O_4_-**1:2** was ·OH > h^+^ > ·O_2_^−^, which is the same trend observed for all nanoparticles against rhodamine B. Fe_3_O_4_-**1.5:2** followed the order ·O_2_^−^ > h^+^ > ·OH, while Fe_3_O_4_-**2:3** follows the sequence of h^+^ > ·O_2_^−^ > ·OH [68,69,70,71]. The addition of MeOH in methyl red reaction media inhibited degradation efficiency from 30.6 to 8.8% (for Fe_3_O_4_-**1:2**), 73.9 to 4.8% (for Fe_3_O_4_-**2:3**), and 47.4 to 6.1% (for Fe_3_O_4_-**1.5:2**). The significant reduction in efficiency suggests that the hydroxyl radicals are major reactive species during the photodegradation. Similarly, the addition of AC reduced the performance of Fe_3_O_4_-**1:2** (9.6–30.6%), Fe_3_O_4_-**1.5:2** (2.9–47.4%), and Fe_3_O_4_-**2:3** (12.8–73.9%), suggesting that ·O_2_^−^ also has a major role in the photocatalytic degradation of methyl red. The results show that Fe_3_O_4_-**1:2** and Fe_3_O_4_-**2:3** follow a reactive species sequence of ·OH > ·O_2_^−^ > h^+^, while Fe_3_O_4_-**1.5:2** follows an ·O_2_^−^ > ·OH > h^+^ trend [72,73].

### 4.2. Proposed Mechanism

**Scheme 1:**
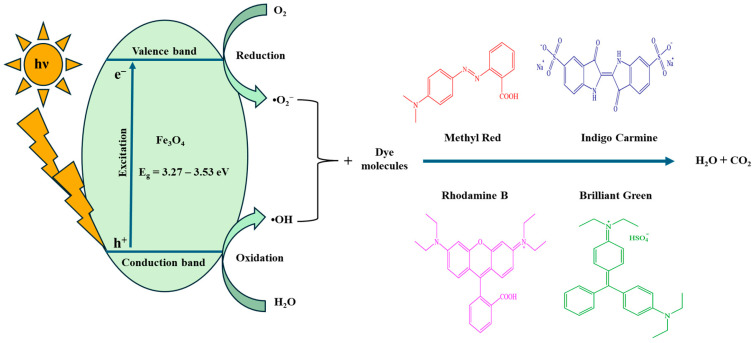
Mechanism of the photocatalytic degradation of MR, IC, RhB, and BG dyes.

The proposed mechanism for the photocatalytic degradation of organic dyes by the as-prepared Fe_3_O_4_ nanoparticles is illustrated in Scheme 1. When the mixture of the dye and iron oxide (Fe_3_O_4_) nanoparticles is subjected to visible light, electron–hole pairs are generated as electrons (e^−^) transition from the valence band to the conduction band. The electrons (e^−^) react with oxygen molecules to generate oxygen peroxide radicals (·O_2_^−^), while the positive charge holes (h^+^) react with H_2_O to produce hydroxyl radicals (·OH). The oxygen peroxide radical undergoes protonation to form hydroperoxyl radicals (·O_2_H) and eventually forms H_2_O_2_, which also dissociates into hydroxyl radicals [55]. The organic dye molecules are degraded by oxidation reactions with ·O_2_^−^ and ·OH radicals, resulting in CO_2_, H_2_O, and some mineralization products.

### 4.3. Effect of pH

**Figure 8:**
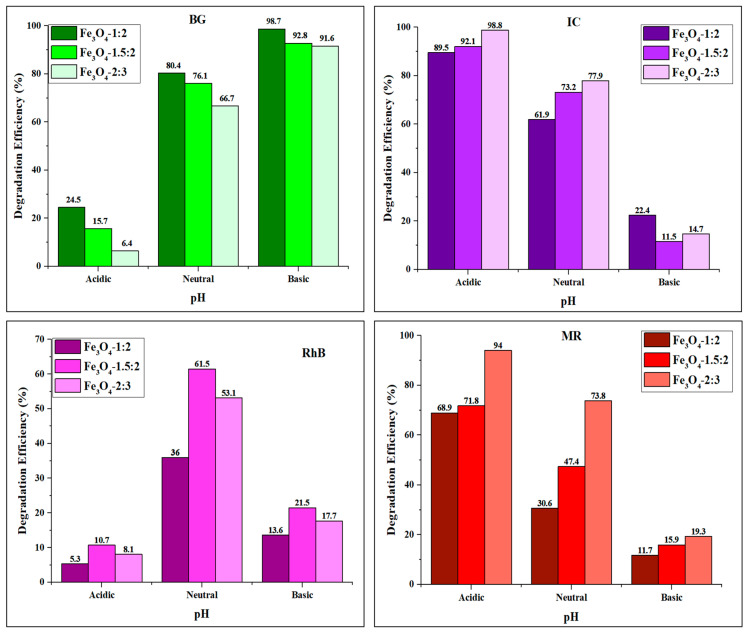
The effect of pH on brilliant green (BG), indigo carmine (IC), rhodamine B (RhB), and methyl red (MR) dyes over Fe_3_O_4_ nanoparticles.

The surface charge of a photocatalyst and the charge of organic dyes are both strongly impacted by pH [74,75,76]. Figure 8 illustrates the findings of a study conducted in neutral, acidic, and alkaline dye solutions to examine the impact of pH on degradation efficiency. The degradation percentage of the BG dye by the iron oxide nanoparticles was lower at a pH of 3 and higher at a pH of 9. The high degradation efficiency observed in the alkaline solution can be attributed to the tertiary carbon of brilliant green, making the dye molecule a carbon-centered electrophile and the excess OH^−^ ions available, making the degradation process more favorable [77]. Rhodamine B degrades most efficiently at neutral pH. When the dye solution is adjusted to either alkaline or acidic, the degradation efficiency is drastically reduced. The dye is cationic (RhB^+^) at pH 3, and high H^+^ concentration reduces the catalyst’s accessible sites for interactions with positively charged RhB dye molecules [78,79]. The RhB^+^ undergoes deprotonation at pH 9 and forms a zwitter ion, which results in the repelling of negatively charged dye molecules and the catalysts due to coulombic forces [80]. A similar phenomenon was observed in other studies using different catalysts [78,81,82]. For indigo carmine, the results show that an increase in the pH of the reaction media decreases the photocatalytic degradation efficiency. At a pH of 3, the highest degradation efficiency was 98.8% after 180 min for Fe_3_O_4_-**2:3**, while at a pH of 9, the lowest degradation of 11.5% for Fe_3_O_4_-**1.5:2** was obtained. Indigo carmine is negatively charged over wide pH ranges, and its structure breaks into hydrocarbon anion fragments with negatively charged sulfonate groups and sodium ions [83,84]. The drastic differences in the degradation efficiency observed over the 3–9 pH range could be due to the coulomb interactions between the surface of the catalysts and the IC dye molecules [85]. Similar results were obtained in the study by Bhakpar et al. using ZnO nanoparticles [75]. The degradation percentage was also found to be higher in an acidic medium than in neutral and alkaline pH for methyl red. Low degradation efficiency (11.7% for Fe_3_O_4_-**1:2**, 15.9% for Fe_3_O_4_-**1.5:2**, and 19.3% for Fe_3_O_4_-**2:3**) were observed at pH 9, while high degradation efficiency of 68.9%, 71.8% and 94.0% for Fe_3_O_4_-**1:2**, Fe_3_O_4_-**1.5:2**, and Fe_3_O_4_-**2:3** were obtained at pH 3. This is mainly because pH has a considerable effect on the molecular structure of methyl red. In an acidic medium, the structure of methyl red transforms into quinoid form, which is unstable and easily degradable. In contrast, the dye molecules in basic media are in the benzenoid form, which is stable and difficult to degrade [86,87]. The findings are consistent with those of previous studies [88,89].

### 4.4. Recyclability of Iron Oxide Nanoparticles

**Figure 9:**
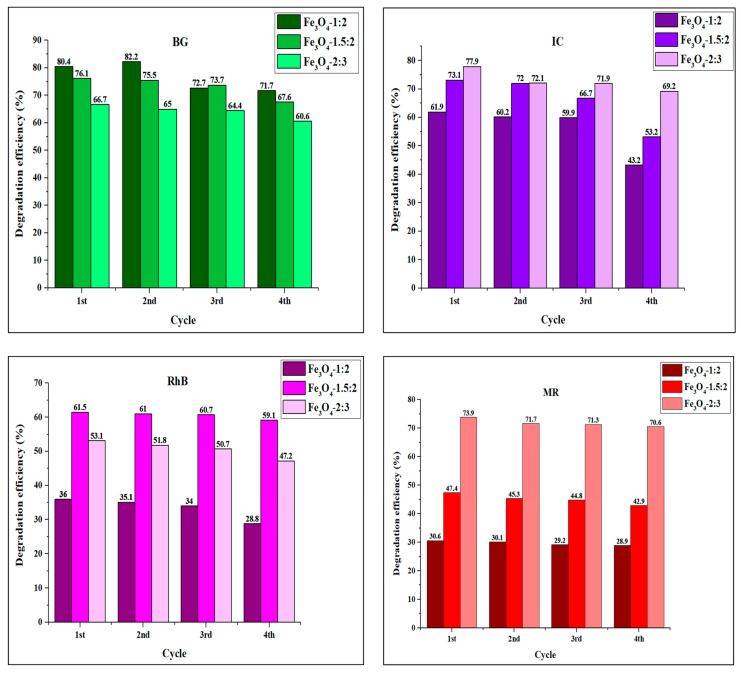
The recyclability of Fe_3_O_4_ nanophotocatalysts.

The stability of the photocatalyst is a significant parameter for its potential practical application in industry [90]. As a result, the recyclability of the Fe_3_O_4_ photocatalysts was assessed in four runs. A degradation efficiency reduction of about 6.1–8.7%, 2.4–7.2%, 8.7–18.7%, and 1.7–4.5% was observed for BG, RhB, IC, and MR, respectively, as shown in Figure 9. This suggests that the as-synthesized iron oxide nanoparticles exhibit good photostability and can be recycled in dye photodegradation reactions (Figures S4–S7). The decrease in degradation percentage may be attributed to the adsorption of the intermediates produced by the degradation of organic dyes onto the active sites of the nanoparticle’s active sites [91].

## 5. Materials and Methods

### 5.1. Materials

FeCl_2_·4H_2_O (≥99%), Fe_2_(SO_4_)_3_·H_2_O (≥97%), ammonia solution (25%), octylamine (≥99%), ethanol (≥96%), brilliant green (≥90%), rhodamine B (95%), methyl red (95%), indigo carmine (~85%), sodium hydroxide (≥98%), HCl (37%), ammonium oxalate (≥99%), acrylamide (99%), and methanol (99.9%) were obtained from Sigma-Aldrich (St. Louis, MO, USA) and were utilized as supplied.

### 5.2. Preparation of Iron Oxide Nanoparticles

Iron oxide nanoparticles were prepared using the co-precipitation approach [92]. Two separate solutions of FeCl_2_·4H_2_O and Fe_2_(SO_4_)_3_·H_2_O were made using 50 mL of water and co-precipitated with 15 mL 25% ammonia solution at 90 °C under nitrogen flow. After adding base to the solution, the reaction mixture was stirred for 30 min; thereafter, 20 mL of octylamine was introduced into the solution, and the resulting mixture was further stirred for 1 h. Three different Fe^2+^:Fe^3+^ mole ratios of 1:2, 1.5:2, and 2:3 were used to prepare Fe_3_O_4_-**1:2**, Fe_3_O_4_-**1.5:2**, and Fe_3_O_4_-**2:3**, respectively. The nanoparticles were separated using a magnet, rinsed with distilled water and ethanol, and then oven-dried at 70 °C for 3 h.

### 5.3. Physical Characterization

Transmission electron microscopy (TEM) micrographs were taken using a JEOL JEM-1400 electron microscope (JEOL, Tokyo, Japan) and Gatan digital microgram software https://www.gatan.com/products/tem-analysis/gatan-microscopy-suite-software accessed on 15 June 2024, and the size distributions were calculated using ImageJ 1.53t software. A Bruker D8 Advance diffractometer (Bruker, Billerica, MA, USA) was employed to record the diffractograms. FTIR spectra were recorded using the Cary 630 FTIR spectrometer (Agilent Technologies, Santa Clara, CA, USA) in the 4000–500 cm^−1^ range. Absorption spectra were recorded on a Perkin Elmer lambda 25 UV–Vis spectrophotometer (PerkinElmer, Waltham, MA, USA) in the 200–700 nm range.

### 5.4. Adsorption Studies

Adsorption studies were conducted by dispersing 5 mg of the as-prepared Fe_3_O_4_ nanoparticles in 5 mL of 10 mg/L dye solution in seven test tubes. The dye–adsorbent mixture was agitated for 90 min with a magnetic stirrer bar. At 15 min intervals, sample aliquots were collected, and a magnet was used to separate the adsorbent from the solution. The dye adsorption was monitored using a UV-Visible spectrometer. The dye adsorption Qt (mg/g) was determined by Equation (3) [93].

Q = ((C_0_ − C_t_)/W) × V (3)

C_0_ represents the initial dye concentration (mg/L), C_t_ represents the dye concentration (mg/L) at the time ‘t’, V represents the dye volume (L), and W represents the adsorbent mass (g).

### 5.5. Photocatalysis Experiment

The photocatalytic potential of the iron oxide nanoparticles was studied by evaluating the degradation of brilliant green (BG), rhodamine B (RhB), indigo carmine (IC), and methyl red. A total of 5 mg of the as-prepared Fe_3_O_4_ nanoparticles was dispersed in 5 mL of 10 mg/L dye solution in seven test tubes. The heterogeneous solution was sonicated for 30 min. and then magnetically agitated in the dark for 60 min to reach adsorption–desorption equilibrium. The dye–photocatalyst mixture was then subjected to an OSRAM VIALOX 70 W mercury lamp (OSRAM, Munich, Germany) with continuous agitation for 180 min at room temperature. At 30 min intervals, 5 mL aliquots were collected, magnet-separated, and the dye was analyzed with a UV-Vis spectrophotometer. The degradation efficiency was determined using Equation (4) [94]:

D = (C_0_ − C_t_)/C_0_ × 100 (4)

D denotes degradation efficiency, and C_0_ and C_t_ denote the dye concentrations at t_0_ and time t, respectively. 

The catalysts were recycled four times to assess their photostability and reusability. Following each run, the catalyst was magnetically removed from the dye solution, rinsed with distilled water and ethanol, and oven-dried at 70 °C for 3 h.

### 5.6. Effect of pH

The effect of pH on the photodegradation of BG, RhB, IC, and MR by Fe_3_O_4_ was investigated at pH 3, pH 6.5, and pH 9. 0.1 M aqueous hydrochloric acid and sodium hydroxide solutions were used to adjust the pH of the dye solutions. A 0.01 M NaCl solution was used for the point of zero charge (pH_zpc_) test for magnetite nanoparticles, which was conducted between the pH values of 2 and 10. Fe_3_O_4_ nanoparticles (0.03 g) were dispersed in 30 mL of NaCl solution and shaken for 24 h at the ambient temperature. The pH of the supernatant was determined. The change in the solution pH was calculated and plotted over the initial solution pH to determine the pH_zpc_. 

### 5.7. Effect of Scavengers

The quenching experiment was used to examine the reactive species responsible for the photocatalytic degradation of the BG, RhB, IC, and MR dyes by the magnetite nanoparticles. Acrylamide (AC), ammonium oxalate (AO), and methanol (MeOH) were used as ·O_2_^−^, h^+^, and ·OH quenchers. Scavengers at a concentration of 10 mM were added to the dye solutions, and the reaction was carried out using the same reaction parameters as the photocatalysis experiment.

## 6. Conclusions

Magnetite iron oxide nanoparticles were synthesized by co-precipitating using three different iron (II) and iron (III) salt mole ratios. The p-XRD patterns correspond to the magnetite crystalline phase of iron oxide. The as-synthesized iron oxide nanoparticles consist of nanoparticles with different mixed shapes with average sizes of 5.48 nm, 6.02 nm, and 6.98 nm for Fe_3_O_4_-**1:2**, Fe_3_O_4_-**1.5:2**, and Fe_3_O_4_-**2:3**. The energy gaps are 3.53, 3.44, and 3.27 eV for Fe_3_O_4_-**1:2**, Fe_3_O_4_-**1.5:2**, and Fe_3_O_4_-**2:3**, respectively. The results show the iron salt ratio influences the morphology and energy band gap of the as-prepared Fe_3_O_4_ nanoparticles. Photocatalytic degradation of BG by the Fe_3_O_4_ nanoparticles after 180 min are 80.4% (Fe_3_O_4_-**1:2**), 76.1% (Fe_3_O_4_-**1.5:2**), and 66.7% (Fe_3_O_4_-**2:3**). Degradation efficiencies of 36.61.5% (Fe_3_O_4_-**1:2**), 61.5% (Fe_3_O_4_-**1.5:2**), and 53.1% (Fe_3_O_4_-**2:3**) were obtained against RhB. While degradation efficiencies of 61.9% for Fe_3_O_4_-**1:2**, 73.2% for Fe_3_O_4_-**1.5:2**, and 77.9% for Fe_3_O_4_-**2:3** were obtained against IC, and the maximum percentage degradation of 73.9% was attained for Fe_3_O_4_-**2:3** against MR. The results of this study revealed that the morphology; ·OH, ·O_2_^−^, and h^+^ reactive species; and pH influence the photodegradation efficiency of BG, RhB, IC, and MR by the as-prepared Fe_3_O_4_ nanoparticles. The as-prepared Fe_3_O_4_ nanoparticles are recyclable and are photostable for the degradation of the organic dyes. Photocatalytic degradation efficiencies of over 70% were obtained against some of the dyes, and their general photostability indicates that the synthetic magnetite iron oxide nanoparticles could be fine-tuned for practical applications.

## Figures and Tables

**Table 1:** Photocatalytic degradation efficiency of magnetite nanoparticles compared to other photocatalysts.

Dye	Catalyst	Method of Synthesis	Size (nm)	Catalyst Load (mg)	Dye Concentration (ppm)	Degradation Efficiency (%)	Time (min)	Light Source	Reference
BG	NiO/CeO_2_	ultrasound	-	200	10	81.9	240	8 W UV light	[48]
	Fe_3_O_4_/ CdS–ZnS	ultrasound	25–45	1.5	10	92.93	360	300 W tungsten lamp	[49]
	SnO_2_/Fe_2_O_3_	hydrothermal	21.41	50	10	74	75	300 W visible light	[50]
	Fe_3_O_4_-1:2	co-precipitation	5.48	5	10	80.4	180	70 W visible light	This study
IC	CoFe_2_O_4_/ SnO_2_	sol-gel/ sonochemical	13	100	5	55	120	15 W UV-A lamp	[51]
	Zr/CuFe_2_O_4_	chemical precipitation	42	100	20	71	120	150 W tungsten lamp	[52]
	ZnFe_2_O_4_	solvothermal	-	50	16	66.3	300	128 W visible light	[53]
	Fe_3_O_4_-2:3	co-precipitation	6.98	5	10	77.9	180	70 W visible light	This study
MR	α-Fe_2_O_3_	co-precipitation	54	100	10	76	5	32 W UV/H_2_O_2_	[54]
	Fe_3_O_4_/ZrO_2_	chemical reduction	803.54 ± 5.11	20	70	84	40	UV-light	[22]
	GO/Fe	in situ	-	0.03	10	70	30	250 W Xenon lamp	[55]
	Fe_3_O_4_-2:3	co-precipitation	6.98	5	10	73.9	180	70 W visible light	This study
RhB	Fe_3_O_4_@SiO_2_@TiO_2_	co-precipitation/Stöber	8–18	50	30	29.49	60	UV 8/18	[56]
	Fe_2_O_3_	co-precipitation	42–49	40	50	58.21	240	Sunlight	[57]
	Fe_2_O_3_	chemical precipitation	3	1410	24.25	38.03	120	202 W visible light	[58]
	Fe_3_O_4_-1.5:2	co-precipitation	6.02	5	10	61.5	180	70 W visible light	This study

**Table 2:** Degradation rate constants of BG, RhB, IC, and MR by Fe_3_O_4_ nanoparticles.

Catalyst	BG		RhB		IC		MR	
	k (min^−1^)	R^2^	k (min^−1^)	R^2^	k (min^−1^)	R^2^	k (min^−1^)	R^2^
Fe_3_O_4_-**1:2**	0.0091	0.9836	0.0024	0.9825	0.0053	0.9925	0.0021	0.9955
Fe_3_O_4_-**1.5:2**	0.0083	0.9942	0.0053	0.9966	0.0073	0.9977	0.0035	0.9961
Fe_3_O_4_-**2:3**	0.0061	0.9797	0.0043	0.9912	0.0084	0.9959	0.0074	0.9987

## Data Availability

All relevant data have been included in the manuscript.

## Supplementary Materials

The following supporting information can be downloaded at https://www.mdpi.com/article/10.3390/ijms25147876/s1.
