# Supplementary material for: Photocatalytic Degradation of Organic Dyes by Magnetite Nanoparticles Prepared by Co-Precipitation
Source: Int J Mol Sci. 2024 Jul 18;25(14):7876. doi: 10.3390/ijms25147876 (PMC11277247; doi:10.3390/ijms25147876)
Supplement: Supplementary file 1 [file ijms-25-07876-s001.zip › ijms-3066825-supplementary.pdf]

Photocatalytic Degradation of Organic Dyes by Magnetite Nanoparticles Prepared by Co-Precipitation

School of Chemistry and Physics, University of KwaZulu-Natal, Private Bag X01, Scottsville, Pietermaritzburg 3209, South Africa; 216006335@stu.ukzn.ac.za

* Correspondence: ajibadep@ukzn.ac.za

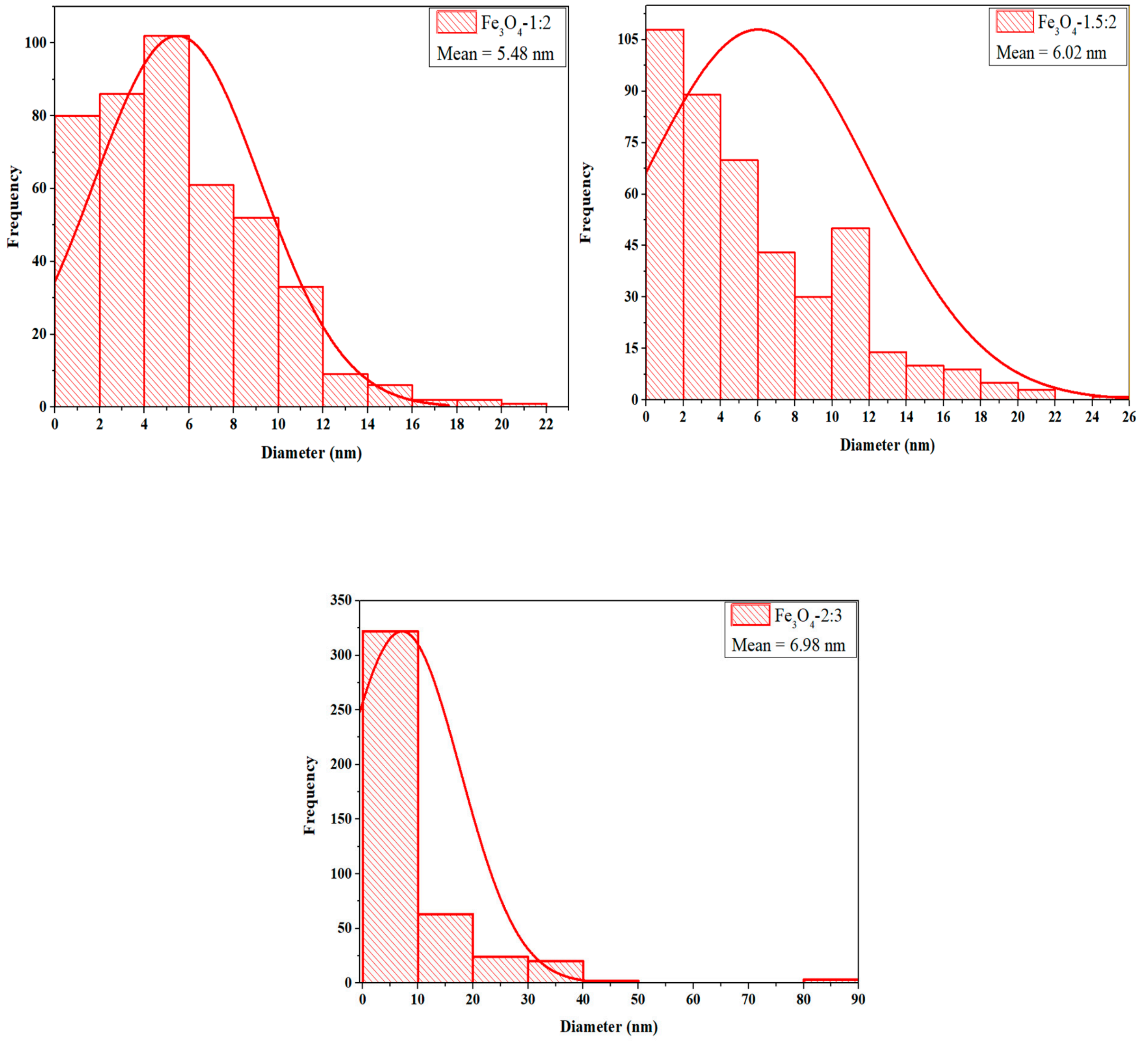

Figure S1: Size distribution histograms of Fe_3O_4-**1:2**, Fe_3O_4-**1.5:2** and Fe_3O_4-**2:3** nanoparticles.

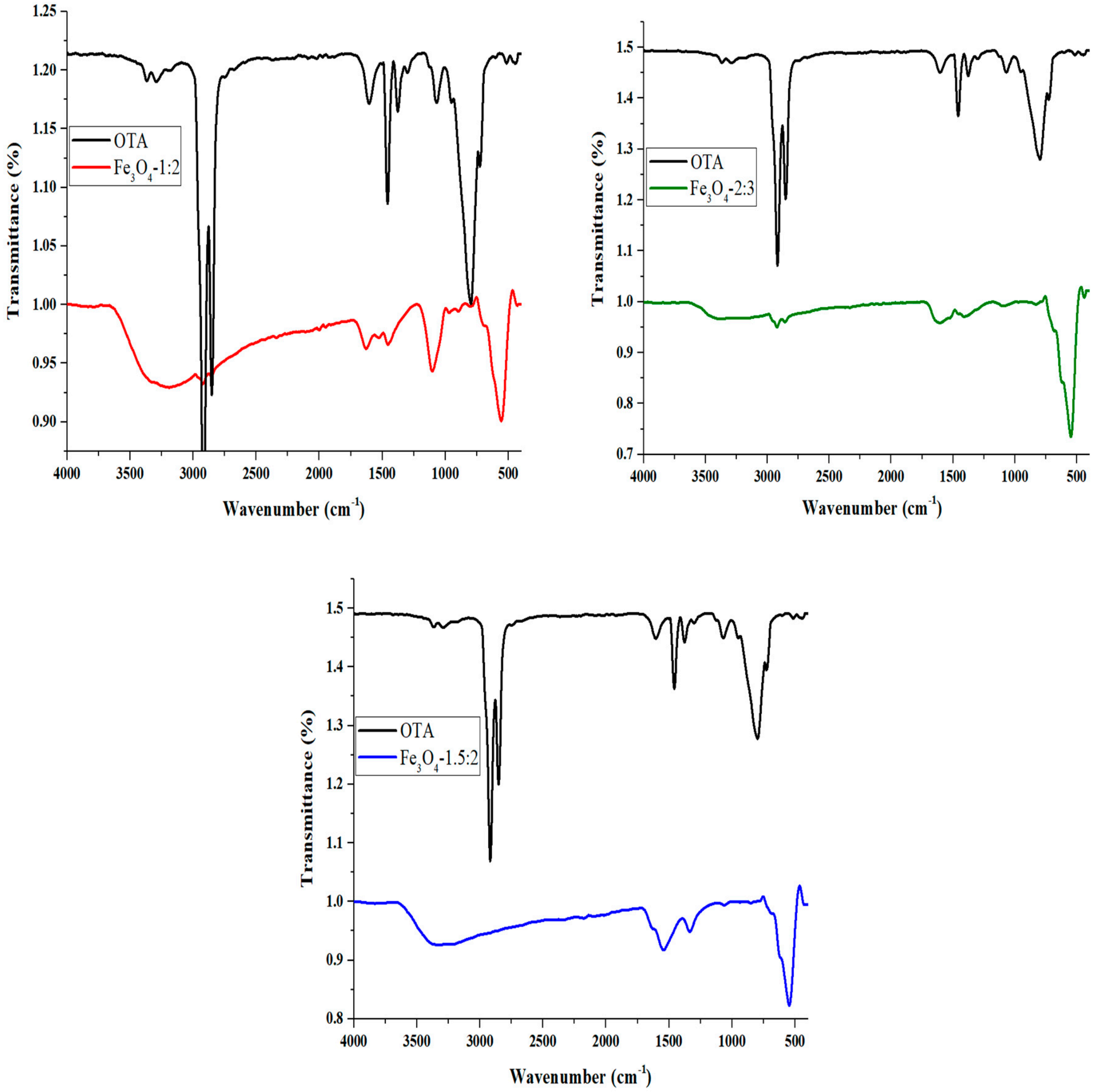

Figure S2. FTIR spectra overlay of Fe_3O_4-**1:2**, Fe_3O_4-**1.5:2** and Fe_3O_4-**2:3** nanoparticles.

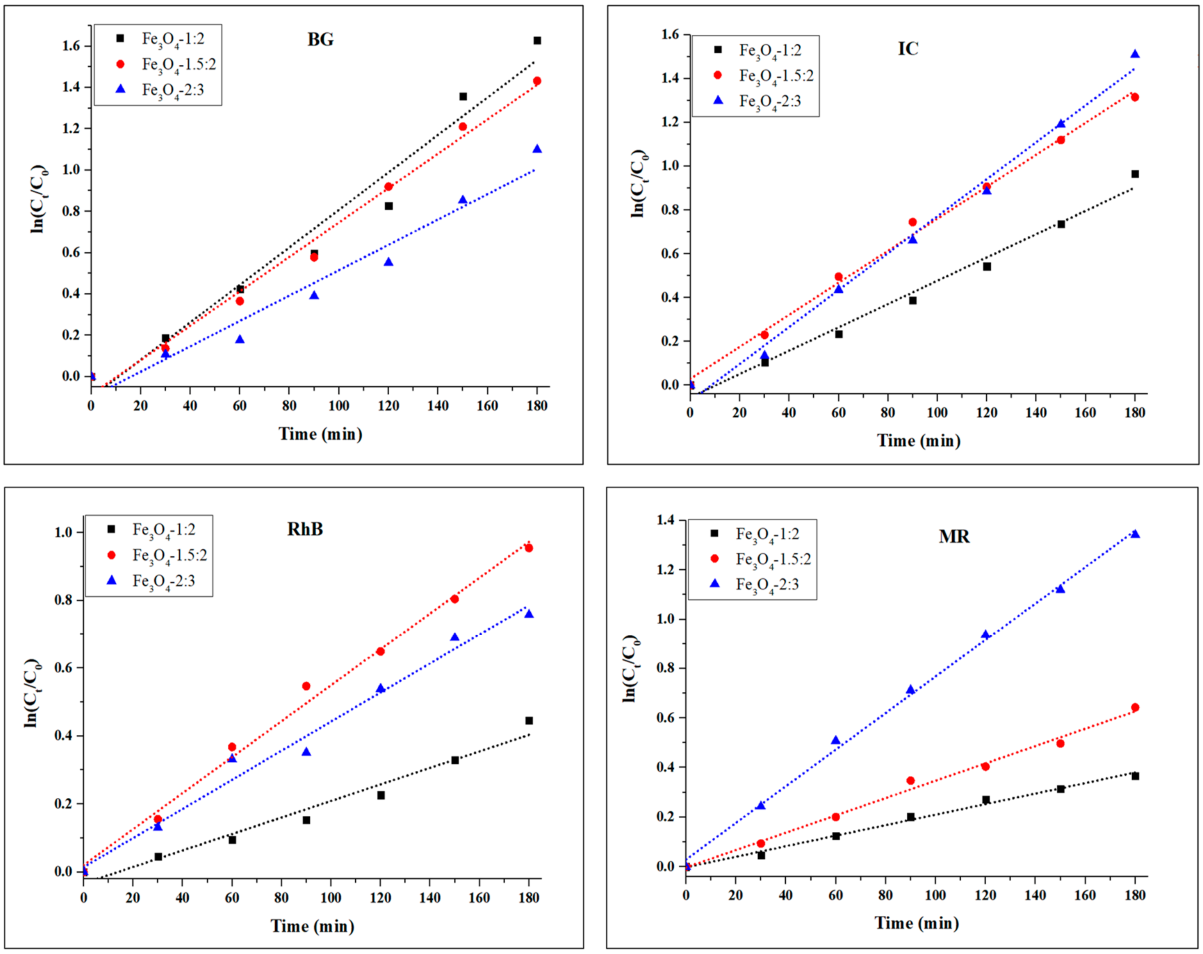

Figure S3. Brilliant green (BG), rhodamine B (RhB), indigo carmine (IC) and methyl red (MR) photocatalytic degradation kinetics using Fe_3O_4 nanoparticles as photocatalysts.

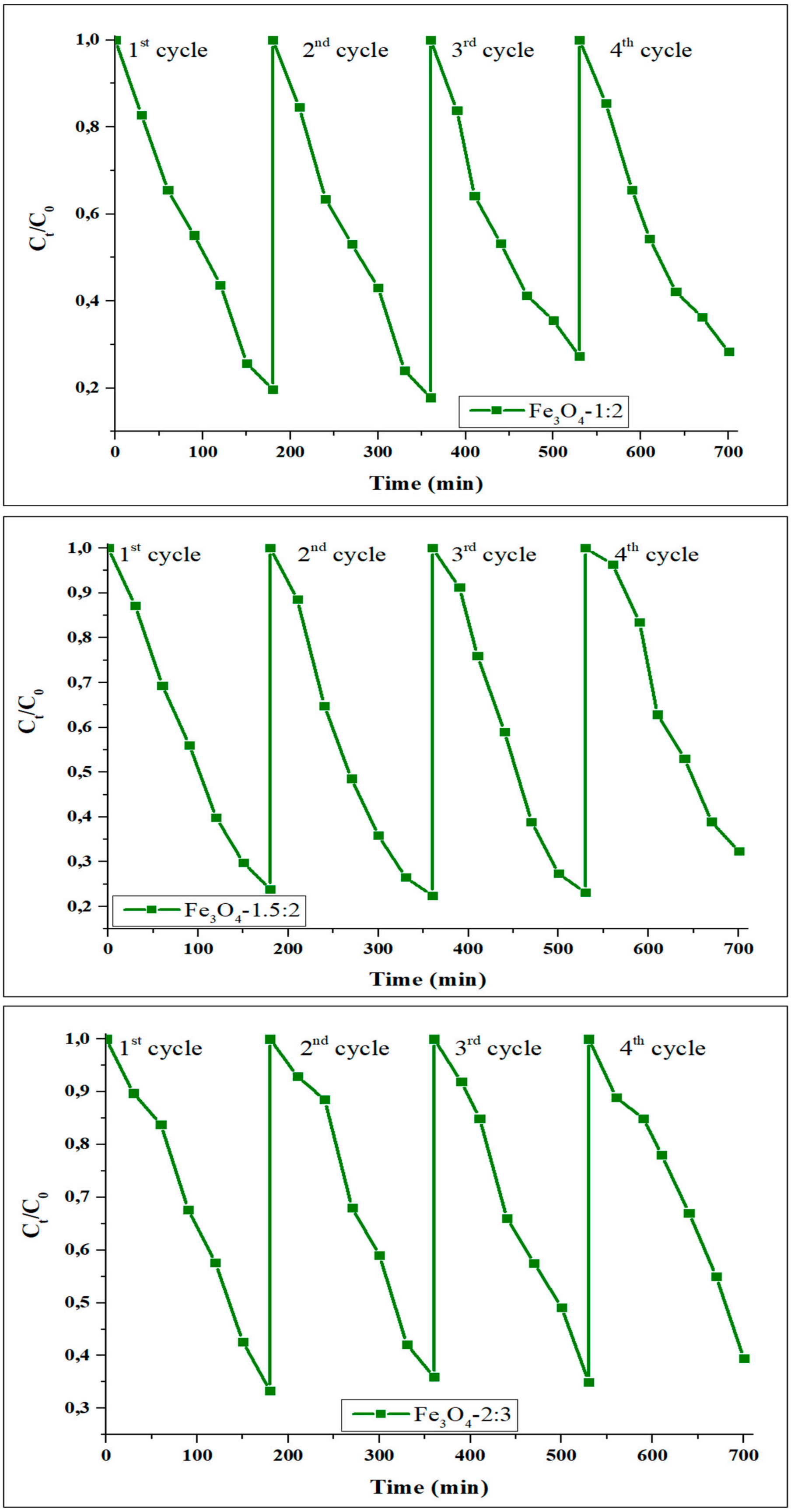

Figure S4: Photostability of Fe_3O_4-**1:2**, Fe_3O_4-**1.5:2** and Fe_3O_4-**2:3** nanoparticles over brilliant green dye.

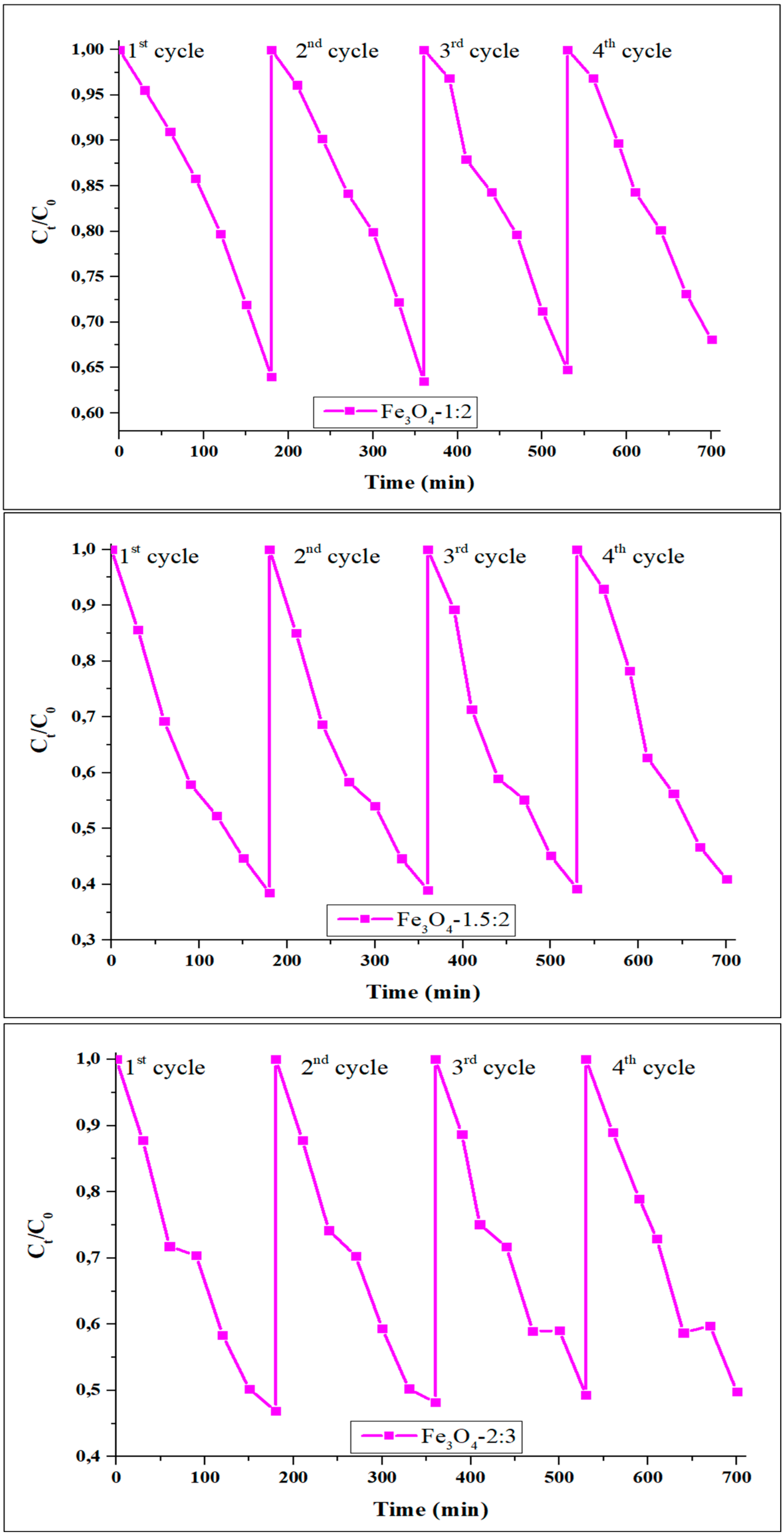

Figure S5: Photostability of Fe_3O_4-**1:2**, Fe_3O_4-**1.5:2** and Fe_3O_4-**2:3** nanoparticles over rhodamine B dye.

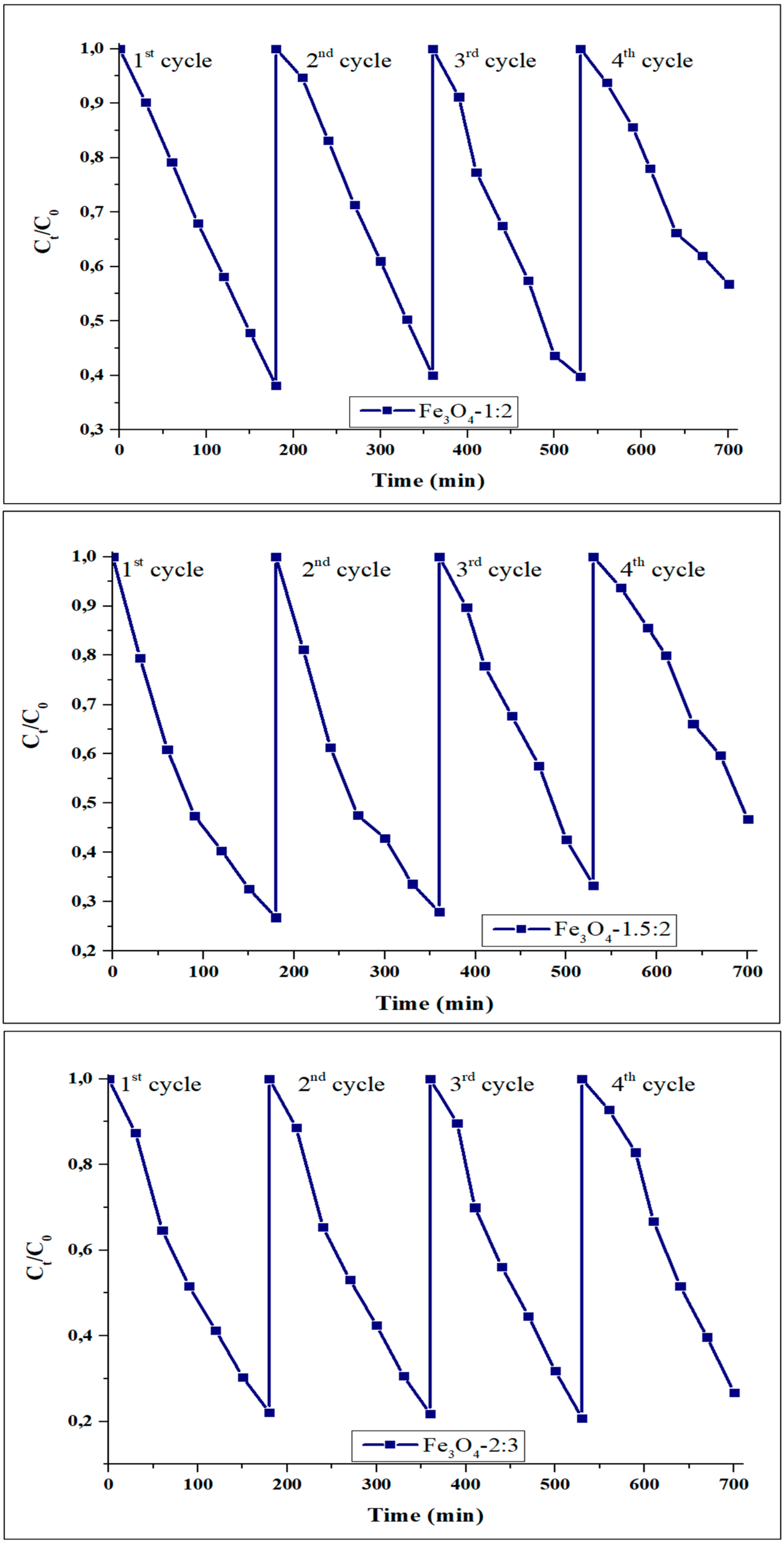

Figure S6: Photostability of Fe_3O_4-**1:2**, Fe_3O_4-**1.5:2** and Fe_3O_4-**2:3** nanoparticles over indigo carmine dye.

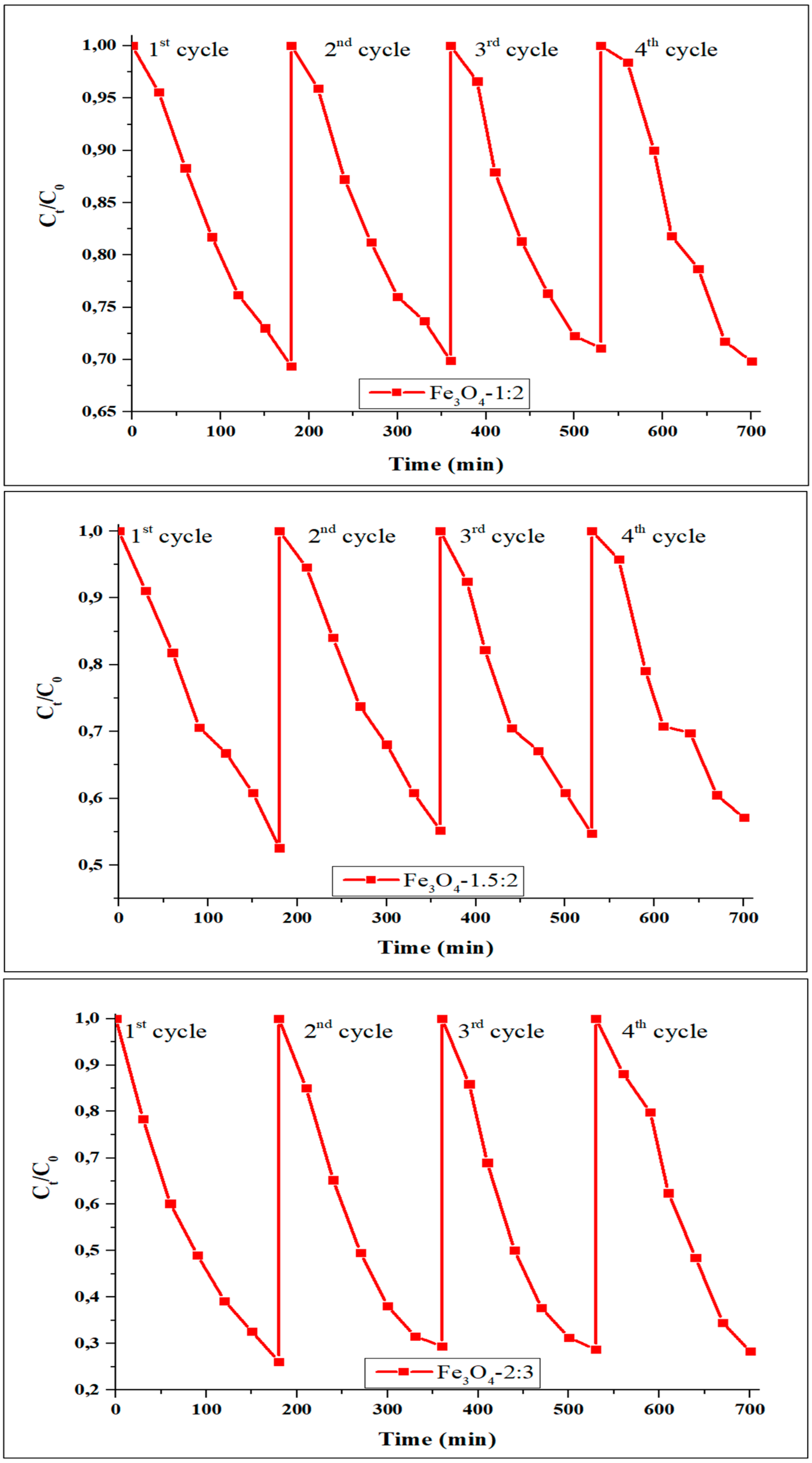

Figure S7: Photostability of Fe_3O_4-**1:2**, Fe_3O_4-**1.5:2** and Fe_3O_4-**2:3** nanoparticles over methyl red dye.
